# Quality of Life, Anxiety, and Depression in Patients With Early-Stage Mycosis Fungoides and the Effect of Oral Psoralen Plus UV-A (PUVA) Photochemotherapy on it

**DOI:** 10.3389/fmed.2020.00330

**Published:** 2020-08-05

**Authors:** Thomas Graier, Regina Fink-Puches, Stephanie Porkert, Roland Lang, Sophie Pöchlauer, Gudrun Ratzinger, Adrian Tanew, Sylvia Selhofer, Paul-Gunther Sator, Angelika Hofer, Alexandra Gruber-Wackernagel, Franz J. Legat, Pablo Augusto Vieyra-Garcia, Franz Quehenberger, Peter Wolf

**Affiliations:** ^1^Research Unit for Photodermatology, Department of Dermatology and Venereology, Medical University of Graz, Graz, Austria; ^2^Department of Dermatology, Medical University of Vienna, Vienna, Austria; ^3^Department of Dermatology and Allergology, Paracelsus Medical University, Salzburg, Austria; ^4^Department of Dermatology, Hietzing Hospital, Vienna, Austria; ^5^Department of Dermatology Venereology and Allergology, Medical University of Innsbruck, Innsbruck, Austria; ^6^Institute for Medical Informatics, Statistics, and Documentation, Medical University of Graz, Graz, Austria

**Keywords:** mycosis fungoides, quality of life, anxiety, depression, PUVA, phototherapy

## Abstract

**Background:** Little is known about psychological discomfort and quality of life (QoL) in early stage mycosis fungoides (MF) and the effect of psoralen plus UV-A (PUVA) on it.

**Objective:** To evaluate QoL, anxiety, and depression with validated instruments in early stage MF patients and whether PUVA treatment improves it.

**Methods:** Patients with stage IA to IIA MF were treated with PUVA twice weekly for 12–24 weeks, followed by maintenance treatment or not, in a prospective randomized clinical trial. Patients completed a questionnaire on DLQI as well as the Hospital Anxiety and Depression Scale (HADS) prior to therapy, after their last PUVA exposure, and after the PUVA maintenance or observance phase.

**Results:** For 24 patients with early stage MF, completed questionnaires were available and analyzed. Prior to treatment, 17% reported strong (DLQI > 10) and 29% moderate impairment (DLQI 6–10) in QoL; 33% of patients reported HADS scores indicating anxiety, and 21% reported scores indicating depression. PUVA significantly improved overall QoL by reducing mean DLQI scores by 58.6% (*p* = 0.003), HADS-A by 30% (*p* = 0.045), and HADS-D by 44% (*p* = 0.002). Improvements in QoL and psychological well-being seemed to be sustained, irrespective of maintenance treatment or not.

**Limitations:** Small sample size.

**Conclusions:** PUVA sustainably improves QoL and psychological well-being in patients with early stage MF.

**Clinical trial registration:** ClinicalTrials.gov identifier: NCT01686594.

## Introduction

Studies show that MF leads to impaired quality of life (QoL) (1–3) and a higher risk for depression and anxiety in affected patients (2, 4). This accounts especially for late-stage MF (2, 5), alopecia within MF lesions, and female gender as a recent study suggests by observing a significantly worse health-related QoL in these patients (2). However, Semenov et al. (6) showed that early stage MF must not be trivialized either as patients with MF stage IA–IIB reported poorer QoL than patients with end-stage kidney disease, diabetes mellitus, or an overall cancer cohort, only to be surpassed by patients suffering from stroke or osteoarthritis. In fact, a nationwide American study on the impact of CTCL on QoL reveals that, overall, 72.7% of CTCL patients felt depressed due to their skin condition, and 39% felt ashamed even though the percentage of early stage MF was 80% in the study (1).

The importance for QoL screening and adequate screening tools in patients suffering from cutaneous lymphomas has recently been highlighted (7). Since MF is commonly considered to be incurable with decreased survival rates in advanced stages (8), disease control, life prolongation, and improvement of quality of life and psychological well-being have emerged as major goals in the treatment of the disease (4, 7, 9, 10). While recent work from the Prospective Cutaneous Lymphoma International Prognostic Index (PROCLIPI) study has enlightened the impact of MF on health-related Qol (2), anxiety and depression have been hardly assessed in clinical trials and, if so, most often using questionnaires not specific and not validated for these conditions. Interest in the potential beneficial effect of therapeutic strategies on patients' QoL increases (11–13), but the effect on psychological comfort remains unknown. This accounts especially for early stage MF and treatment with phototherapy. So far, the impairment of QoL in phototherapeutically treated patients has only been investigated in patients treated with photopheresis (14).

Phototherapy belongs to the most frequently used therapeutic approaches in early stage disease (15), of which psoralen plus UVA (PUVA) photochemotherapy is considered to be a very efficient and well-tolerated treatment option despite its potential cancerogenic effects (10, 16). In fact, PUVA increases the risk for non-melanoma skin cancer, mainly squamous cell skin cancer, in a dose-dependent fashion (i.e., at >200 sessions or cumulative dosage of 2,000 J/cm^2^) (17). Furthermore, a timely delayed increased risk for melanoma has been observed in patients exposed to more than 200 PUVA irradiations 15 years after the first PUVA treatment (18, 19). However, in terms of efficacy, PUVA with its complete response rate of 70% in this study population (20) beats, by far, other treatment options, such as bexarotene (complete response rates 7–13%) (21, 22) or interferon α-2a plus acitretin (complete response rate 38%) (23). Furthermore, a recent meta-analysis reveals that complete response rates of PUVA being 73.8% in early stage MF (Ia-IIa) were significantly superior to that of UVB with 62.2% (24). Moreover, PUVA is considered to be the most helpful treatment option in early stage MF from the patients' perspective (1) although systemic treatments have recently been questioned to improve QoL (25). We, therefore, aimed to evaluate the effect of PUVA on QoL, anxiety, and depression in patients with early stage MF.

## Methods

### Study Design and Setup

The aims of this analysis were the determination of QoL impairment and the psychological burden in patients suffering from early stage MF and the effect of oral PUVA on them. The objectives of the analysis relate to the secondary endpoints of the Austrian trial on low-dose, low-frequency oral psoralen-UV-A treatment with or without maintenance in early stage MF (20, 26). Please see our previously published work for details about the inclusion and exclusion criteria, previous treatments, phototherapeutic characteristics, clinical treatment response, and adverse events (20). This analysis is in accordance with the ethical approval of the Medical University of Graz and in full compliance with the Austrian Medicinal Products Act and in accordance with the International Conference on Harmonization Good Clinical Practice guideline (27). All participants gave written informed consent according to the principles of the Declaration of Helsinki (28).

### Questionnaires

German versions of questionnaires on Dermatology Life Quality Index (DLQI) and Hospital Anxiety and Depression Scale (HADS) were used in this trial. The items of the original study questionnaires are enclosed in the supplements (Supplemental Methods 1).

### Statistical Analysis

The Wilcoxon signed-rank test was applied to determine statistical significance of improvement comparing overall DLQI and HADS scores and single items of the respective questionnaires that had been collected prior to, during, and after PUVA. Fisher's exact test was performed to test for differences in patient characteristics of complete and partial responders. Spearman analysis was performed for evaluation of the correlation between clinical response (mSWAT reduction) and response of QoL and anxiety and depression (DLQI and HADS). Mann-Whitney *U*-test was done for analysis of individual reductions (at the end of PUVA induction) in overall DLQI, HADS-A, and HADS-D comparing patients with complete and partial remission as well as in individual reductions in patients receiving PUVA maintenance therapy or not. Statistics were performed using SPSS V25.0 (IBM Corp. Armon, NY). Graphics were designed with Microsoft Office 365 (Microsoft Corporation, Redmond, USA) and Adobe Acrobat DC Pro V1.7 (Adobe, San Jose, USA). Statistical significance was set at *p* < 0.05.

## Results

### Study Participants

Twenty-seven patients were enrolled in the study of whom one was excluded due to therapy-related adverse events (vomiting and recurring nausea) and missing questionnaires at baseline. Questionnaires on DLQI and HADS were completed prior to and after induction therapy by 24 of the remaining 26 patients enrolled at the five study centers across Austria (Graz, Hietzing, Vienna, Salzburg, Innsbruck) in the trial and available for analysis (for excluded patients, see Supplemental Figure 1). The mean age of the remaining 24 patients (11 stage IA, 12 stage IB, and 1 stage IIA) was 60 (range 30–80) years (Table 1).

**Table 1:** Patient characteristics.

	**Complete responders**	**Partial responders**	***p*-value**
Number of patients	18	6	
Age, mean (range)	57.9 (30–80)	62.3 (31–75)	*p* = 0.542
Sex			
Male	12 (67%)	4 (67%)	*p* = 1.000
Female	6 (33%)	2 (33%)	
Lesion type			
Patch	18 (100%)	6 (100%)	*p* = 0.15
Plaque	5 (28%)	4 (67%)	
Stage			
IA	8 (44%)	3 (50%)	*p* = 0.276
IB	10 (56%)	2 (33%)	
IIA	0 (0%)	1 (17%)	
Initial mSWAT score, mean (range)	17.8 (1–66)	20.85 (5–46)	*p* = 0.700
Comorbidities			
Complete responders	Previous history of penis carcinoma 1, previous history of colon carcinoma 1, Huntington‘s disease 1, coronary heart disease 3, benign prostatic hyperplasia 2, omarthrosis 2, gonarthrosis 1, disc prolapse 2, carpal tunnel syndrome 1, diabetes 1, cholecystolithiasis 2, nephrolithiasis 1, hypertension 4, chronic sinusitis 1		
Partial responders	Previous history of prostate cancer 1, diabetes 1, spinal stenosis 1, obesity 1, gonarthrosis 1, chronic cholecystitis 1, atrial fibrillation 1, hypertension 3, depression 1, hyperlipidemia 1, hypothyreosis 1		
Number of patients having no comorbidities	4/18	1/6	*p* = 1,000

### DLQI

**Figure 1:**
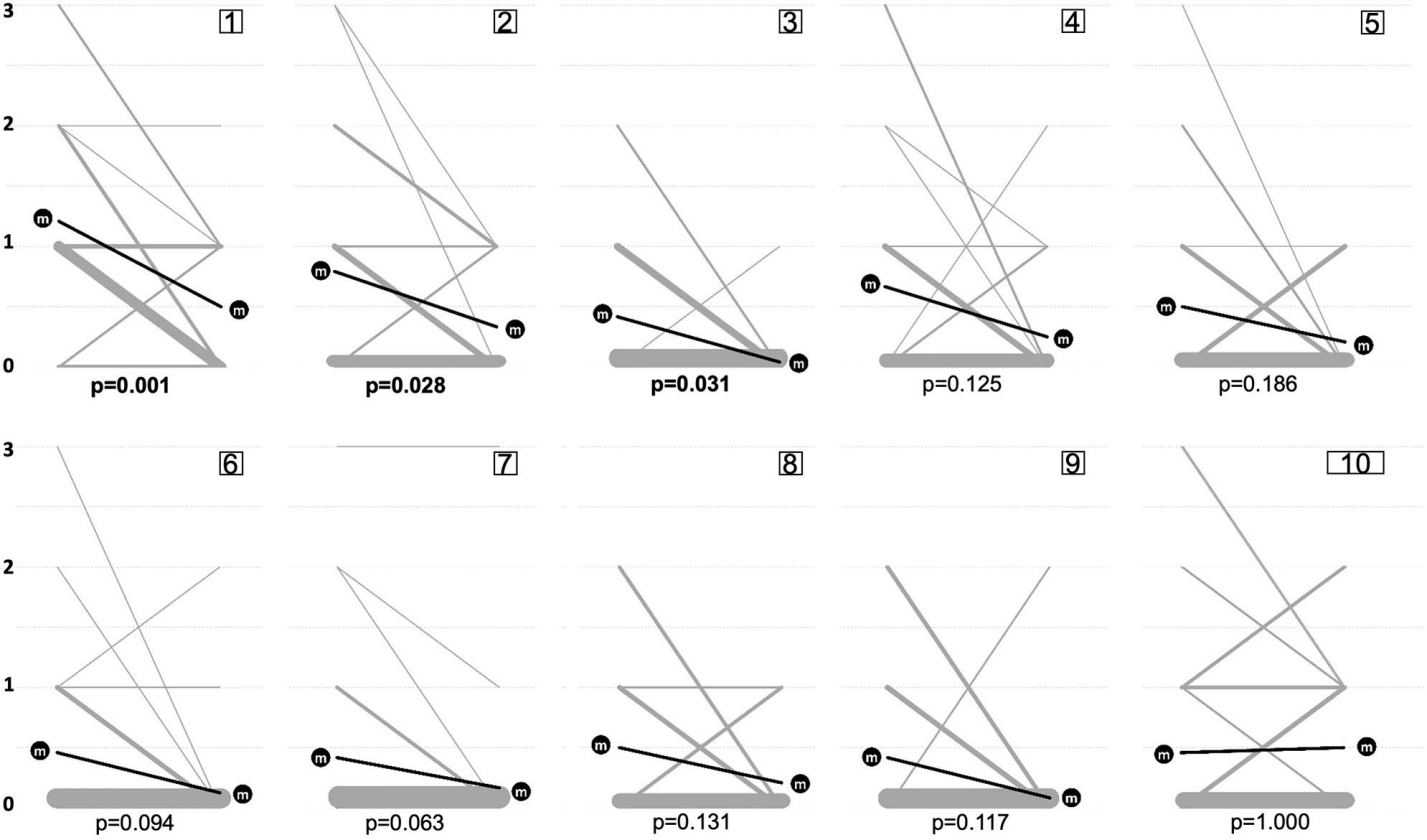
Evolution of DLQI items. Individual values of the 10 Dermatology Life Quality Index (DLQI) items for the time points prior to (left side) and after (right side) Psoralen-UV-A (PUVA) treatment. Numbers in square boxes on top of the panels represent item number of DLQI: item 1 (itching, aching, stinging), item 2 (embarrassment), item 3 (shopping, home), item 4 (affection on clothing), item 5 (social activities), item 6 (sports), item 7 (working, studying), item 8 (interpersonal problems), item 9 (sexual difficulties), and item 10 (treatment difficulties). Each item can be categorized with zero to three points. Items marked as not relevant were awarded with zero points. Thickness of gray lines is proportional to the number of patients and their DLQI evoluting in a certain way. Statistical comparison was performed by Wilcoxon signed-rank test, and respective *p*-values are shown underneath plots. Evolution of mean value (*m*) is shown by the black line and the black circles.

**Figure 2:**
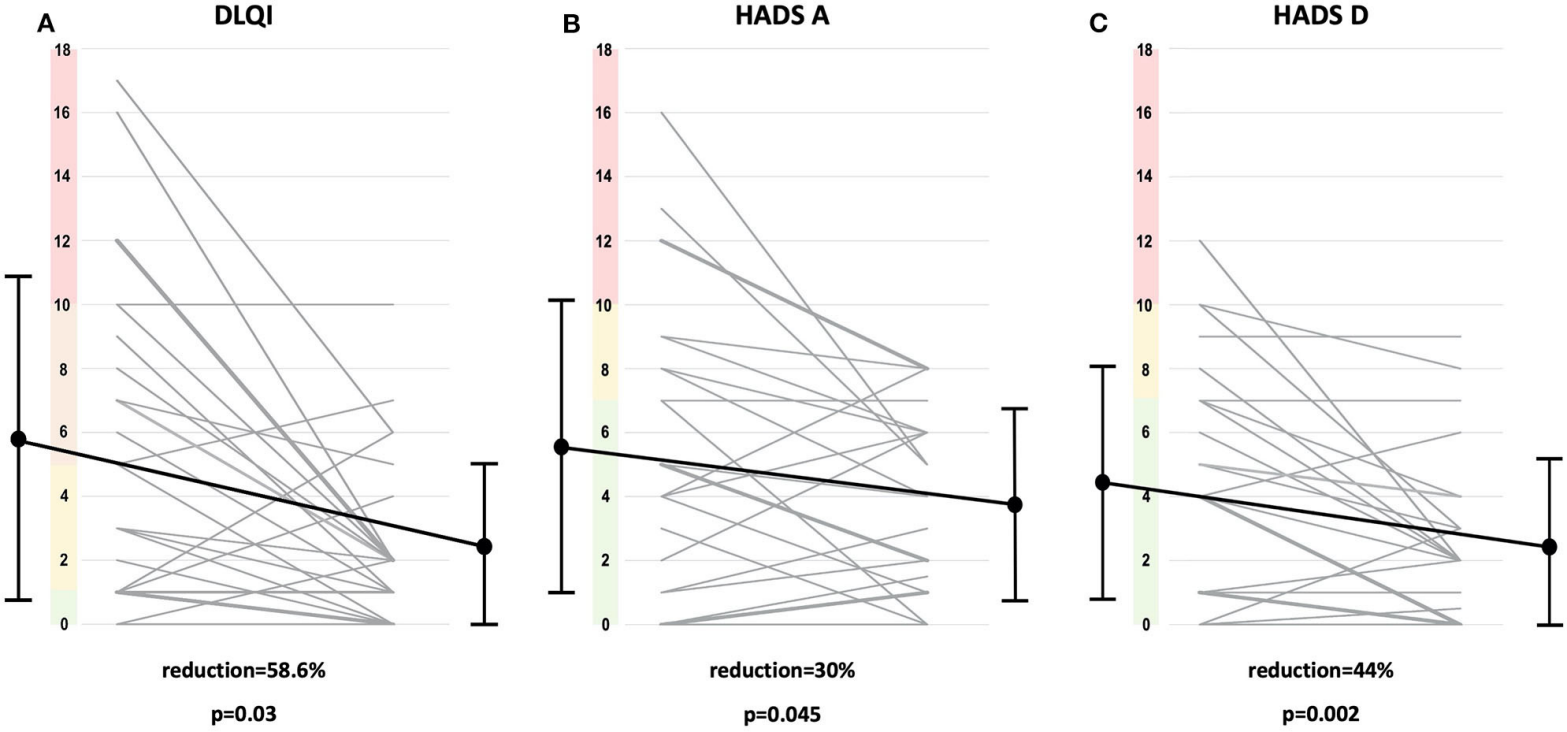
DLQI and HADS scores. Individual overall values of Dermatology Life Quality Index (DLQI) **(A)** and the Hospital Anxiety and Depression scale HADS-A **(B)** and HADS-D **(C)** of the 24 patients prior to (left side) and after psoralen-UV-A (PUVA) induction (right side) and their specific evolution (gray line) are shown. Thickness of gray lines is proportional to the number of patients and their DLQI, HADS-A, or HADS-D evoluting in a certain way. Range of DLQI severity groups are colorized on the *y*-axis of the plot: no impairment (green; 0–1 points); slight (yellow; 2–5), moderate (orange; 6–10), and severe (red; >10 points) impairment. Range of HADS severity groups colorized on the *y*-axis of the plots: no signs of anxiety/depression (green; 0–7 points), borderline abnormal (yellow; 8–10), abnormal values (red; >10 points). Mean values (black dots), standard deviations (black intervals), and evolution of means (black lines) prior to (left side) and after (right side) PUVA treatment are plotted. Percentages of reduction comparing overall scores prior to and after PUVA treatment are depicted. Statistical significance was determined by Wilcoxon signed-rank test and respective *p*-values are plotted.

Prior to treatment, 7 of 24 (29%) patients felt no impairment of QoL at all (DLQI 0-1); 4/24 (17%) reported strong impairment (DLQI >10), 7/24 (29%) moderate impairment (DLQI 6–10), and 6/24 (25%) slight impairment (DLQI 2–5) of QoL by MF (Supplemental Figure 2). Specific DLQI results are shown in Figure 1. Severity of QoL impairment shows no correlation with the degree of affected body surface (Supplemental Figure 3). PUVA led to a significant improvement in severity of QoL affection by reducing overall DLQI scores from a mean of 5.83 ± 4.92 to 2.41 ± 2.56 (58.6% reduction, *p* = 0.003) (Figure 2) and reducing the scores of the most strongly impaired items [i.e., item 1 regarding “itching, aching, or stinging of the skin” (from mean of 1.21 ± 0.82 to 0.50 ± 0.58; *p* < 0.001), item 2 regarding “embarrassment” (from mean of 0.79 ± 0.96 to 0.33 ± 0.47; *p* = 0.028), and item 3 regarding “interfering with daily life routine (e.g., shopping, home, gardening)” (from mean of 0.42 ± 0.64 to 0.04 ± 0.20; *p* = 0.031)]. Subgroup analysis revealed DLQI improvement in complete responders and partial responders without statistically significant differences (Supplemental Table 1). Medical records revealed no significant differences in patient characteristics regarding age and comorbidities of any type between complete and partial responders (Table 1). Taken together, the non-significant specific items (items 4–10) of the DLQI questionnaire (and excluding the significant items 1–3), statistically significant improvement was also reached (*p* = 0.039) (Supplemental Table 2). As depicted in the supplements (Supplemental Table 3), improvement of QoL and anxiety and depression seemed to be sustained at least until the last visit before recurrence, considering the first 9 months after end of induction, irrespective of whether patients were allocated to maintenance or no maintenance treatment (observation arm) (Supplemental Table 3).

### HADS-Anxiety

Prior to phototherapeutic treatment, 16 of 24 (67%) patients felt no signs of anxiety at all, and 8/24 (33%) of the patients reported scores indicating anxiety, consisting of four patients with a borderline score and four patients with an abnormal HADS-A score (Supplemental Figure 2). Severity of anxiety showed no correlation with the degree of affected body surface (Supplemental Figure 3). PUVA led to a significant improvement of overall HADS-A values by improving it from a mean of 5.62 ± 4.56 to a mean of 3.93 ± 2.83 (*p* = 0.045) (Figure 2). After PUVA, four patients showed borderline abnormal HADS-A scores, and normal scores were observed for the remaining patients. Items with the strongest impairments were item 2 “frightened feelings” (15 patients), item 1 “inner tension” (15 patients), and item 6 “restlessness” (16 patients). PUVA led to a significant reduction of patient-reported “inner tension” from a mean of 1.00 ± 0.93 to 0.42 ± 0.50 (item 1; *p* = 0.011) (Supplemental Figure 4). Subgroup analysis failed to show a significant difference in the reductions of HADS-A, comparing complete and partial responders (Supplemental Table 1). Similar as for the DLQI, the improvement of anxiety seems to be sustained (Supplemental Table 3).

### HADS-Depression

At study enrollment, 19 of 24 (79%) patients felt no signs of depression, and 4/24 (17%) patients had a borderline score, and 1/24 (4%) patient an indicative score for depression as reported in the HADS-D questionnaire (Supplemental Figure 2). The results on the specific items of HADS are depicted in Supplemental Figure 4. Severity of depression shows no correlation with the degree of affected body surface (Supplemental Figure 3). PUVA led to a significant improvement of the means in overall HADS-D score from 4.50 ± 3.64 to 2.50 ± 2.65 (*p* = 0.002) (Figure 2) as well as in the specific item score “enjoyment of things” from 0.79 ± 0.78 to 0.33 ± 0.48 (item 1; *p* = 0.016) and in the item “looking forward with enjoyment” from 1.00 ± 1.06 to 0.42 ± 0.77 (item 6; *p* = 0.014). After PUVA, two patients showed borderline abnormal HADS-D scores although normal scores were observed for the remaining patients. Similar as for DLQI and HADS-A, the improvement of depression (as measured by HADS-D) seemed to be sustained. Relative differences comparing changes in DLQI and HADS-A in the course of time after the end of induction treatment ranged between 8.3 and 8.5% for DLQI and −4.8 and 5.7% for HADS-A, respectively, in patients treated with maintenance therapy vs. patients in the observance arm (non-significant). There were larger differences in the HADS-D score, but they did not reach significance (Supplemental Table 3).

### Correlation Analysis

Spearman analysis revealed a statistically significant correlation between DLQI and HADS-A (*r* = 0.518; *p* = 0.009) and HADS-A and HADS-D (*r* = 0.643; *p* = 0.001), analyzing baseline values at start of treatment (Supplemental Table 4 and Supplemental Figure 3). However, no significant correlation between DLQI and HADS-D was observed (*r* = 0.342; *p* = 0.102). Furthermore, overall DLQI improvement after PUVA treatment correlated with HADS-A improvement (*r* = 0.541; *p* = 0.006) but not HADS-D improvement (data not shown). DLQI, HADS-A, and HADS-D did not correlate with mSWAT. Absolute and relative values of improvement after therapy in DLQI, HADS-A, and HADS-D did not correlate with mSWAT either (data not shown).

## Discussion

DLQI scores reported in this study prior to photochemotherapy are in the range of overall scores observed for severe psoriasis (29) and atopic dermatitis (30) although the mean overall score in MF appears to be slightly lower. PUVA improved QoL significantly by reducing the overall DLQI score (Figure 2) and leading to a significant decrease in “skin sensation” (item 1), “embarrassment” (item 2), and “interfering with daily life activities” (item 3) (Figure 1). There have been only a few studies in which the effect of treatment on QoL in MF has been investigated, but none address psychological well-being (11–13, 21, 31). Notably, this work addresses for the first time the effect of photochemotherapy on QoL and psychological comfort in early stage MF. A recent study suggests adjusting overall DLQI scores for answers marked as not relevant and coins the term DLQI-R (32). In our study, the percentages of not relevant marked items was relatively low, and although the calculation of a DLQI-R did slightly increase the significance level of *p*-values, overall, it did not change the results (data not shown). Improvement of QoL using oral psoralen and daylight has been described for patients with severe psoriasis experiencing a higher QoL impairment (33) than the MF patients of this study. Of note, in psoriasis and atopic dermatitis, impairment, and improvement of QoL are linked to body surface extension of disease (34–38). Moreover, psoriasis patients with therapy-induced improvement but not complete clearance of skin may still suffer from substantial QoL impairment (34). However, data on the impact of PUVA in other chronic diseases are limited, making it hard to compare the outcome with our results. In a recently published study (36), narrowband UVB phototherapy decreased overall DLQI values by roughly 63% in patients with psoriasis and by 47% in patients with atopic dermatitis—both sets of patients having higher DLQI values at baseline than the MF patients of this study. The QoL improvements were sustained for at least 3 months after phototherapy end although to a higher extent in the atopic dermatitis than psoriasis patients (36).

The screening and surveillance of anxiety and depression using HADS has not been applied in MF previously although it is reported that the early stage of the disease leads to psychological discomfort (1, 3). HADS has emerged as a reliable instrument for detecting states of depression and anxiety since its publication in 1982 (39). It has been used in dermatology for patients with psoriasis, atopic dermatitis, acne, and hidradenitis suppurative (40–44) and has also been widely accepted as a screening and surveillance tool in non-dermatologic diseases (45–49). The expanded use of HADS allows us to compare our results with that in other dermatologic diseases and beyond. HADS-A and HADS-D scores observed for MF in this study are in the range of scores reported for severe psoriasis (29) and are partly higher than in patients with malignant melanoma stage Ia (50). In comparison with non-dermatologic diseases, the HADS results of this study are in the range of values observed in adolescents with severe asthma, coronary arteria disease, or dialysis (51–53). Strikingly, PUVA led to a significant decrease in overall HADS-A and HADS-D (Figure 2). Similar to QoL, the improvement of psychological comfort seems to be sustained, irrespective of whether patients received maintenance treatment or not (Supplemental Table 3) although (due to small sample size of subgroups) the study was not powered enough to determine statistical significance for this comparison.

Since HADS has not been used in MF before, direct comparison with previous findings remains difficult. However, considering the fact that a higher risk for depression has been reported in MF (3, 4), it is high time for the use of a validated instrument to detect anxiety and depression in such patients. Previous studies use mainly health-related QoL questionnaires to detect psychological discomfort, lacking valid screening of anxiety, and depression (1, 2). Further studies will have to prove if HADS is the adequate instrument for detection of anxiety and depression in early stage MF patients.

We find a statistically significant correlation between DLQI and HADS-A as well as HADS-A and HADS-D, analyzing baseline values at start of treatment (Supplemental Table 4). Notably, DLQI, HADS-A, and HADS-D do not correlate with mSWAT. Improvement in DLQI, HADS-A, and HADS-D after induction treatment does not correlate with mSWAT either. This indicates that patients with early stage MF are affected in QoL and psychological well-being irrespective of extent of disease (as measured by mSWAT). Similar findings were recently observed in a larger cohort of patients with early stage MF although with different QoL instruments (5). In fact, for patients with higher stage MF (>IIA) overall a worse QoL was recently reported (2, 5), indicating that disease stage *per se* does affect QoL more than area and extent of body involvement. A previous study reveals (1) that >93% of the surveyed MF patients worried about their disease being serious and >80% worried about dying. When patients were asked about their treatment, 93% reported good communication about their disease and progress, and 85% of them considered their disease after treatment more manageable than before. Furthermore, 84% were satisfied with the explanations about the indolent course of the disease, possibly helping them to handle their disease (better). Moreover, at least theoretically, (P)UVA may have contributed independently to improvement of psychologic well-being by an opioidergic effect, resulting from UV-induced production of endorphins and its (systemic) release, modulating itch and pain in the skin and reducing patient's stress by interaction with the neuroendocrine system (54–57) as low-level UV was recently reported to induce the production and release of endocannabinoids (58).

## Limitations

The major limitations of this study are the small overall sample size and, from a statistical point of view, the overall good response of all patients without any poor responders. The fact, that we were unable to detect a correlation in reduction of absolute mSWAT values with DLQI and HADS may have been at least additionally hampered by the statistical limitations resulting from low scattering with the high rate of complete responders (with mSWAT values of zero) and partial responders (with mSWAT values near to zero).

## Conclusions

Improvement of quality of life as well as reduction of anxiety and depression are in the spotlight of desirable treatment achievements. This study confirms relatively high impairment of QoL and psychological comfort in patients with early stage MF and discloses the effect of photochemotherapy on it. Sustained improvement of QoL and psychological well-being were linked to PUVA treatment.

## Data Availability Statement

The raw data supporting the conclusions of this article will be made available by the authors, without undue reservation.

## Ethics Statement

The studies involving human participants were reviewed and approved by Ethikkommission der Medizinischen Universität Graz, LKH-Universitätsklinikum—Eingangsgebäude, Auenbruggerplatz 2, 3.OG, A-8036 Graz, ethikkommission@medunigraz.at. The patients/participants provided their written informed consent to participate in this study.

## Author's Note

Parts of this study were presented as poster at the EORTC-CLTF meeting 2019 September 26–28, 2019 in Athens, Greece. We thank Honnavara N. Ananthaswamy, Houston, TX, for critical reading and editing of the manuscript.

## Author Contributions

TG, PV-G, and PW had full access to all of the data in the study, take responsibility for the integrity of the data, the accuracy of the data analysis, concept, and design. PW: supervision and obtained funding. PV-G, RF-P, SPor, SPöc, RL, SS, P-GS, AH, AG-W, FL, and PW: administrative, technical, or material support. TG, FQ, and PW: statistical analysis. TG and PW: drafting of the manuscript. All authors: critical revision of the manuscript for important intellectual content, acquisition, analysis, or interpretation of data.

## Conflict of Interest

The authors declare that the research was conducted in the absence of any commercial or financial relationships that could be construed as a potential conflict of interest.

## Abbreviations

MF: mycosis fungoides
PUVA: psoralen plus UV-A
DLQI: Dermatology Life Quality Index
HADS: Hospital Anxiety and Depression Scale
QoL: quality of life
EORTC: The European Organisation for Research and Treatment of Cancer.

## References

[1] Demierre MF Gan S Jones J Miller DR. Significant impact of cutaneous T-cell lymphoma on patients' quality of life: results of a 2005 National cutaneous lymphoma foundation survey. Cancer. (2006) 107:2504–11. 10.1002/cncr.22252 17048251

[2] Molloy K Jonak C Woei-A-Jin FJSH Guenova E Busschots AM Bervoets A. Characteristics associated with significantly worse quality of life in mycosis fungoides/Sézary syndrome from the prospective cutaneous lymphoma International prognostic index (PROCLIPI) study. Br J Dermatol. (2019) 182–770–9. 10.1111/bjd.18089 31049926

[3] Hodak E Lessin S Friedland R Freud T David M Pavlovsky L. New insights into associated co-morbidities in patients with cutaneous T-cell lymphoma (mycosis fungoides). Acta Derm Venereol. (2013) 93:451–5. 10.2340/00015555-1496 23303582

[4] Sampogna F Frontani M Baliva G Lombardo GA Alvetreti G Di Pietro C. Quality of life and psychological distress in patients with cutaneous lymphoma. Br J Dermatol. (2009) 160:815–22. 10.1111/j.1365-2133.2008.08992.x 19120325

[5] Herbosa CM Semenov YR Rosenberg AR Mehta-Shah N Musiek AC. Clinical severity measures and quality of life burden in patients with mycosis fungoides and Sézary syndrome: comparison of generic and dermatology-specific instruments. J Eur Acad Dermatology Venereol. (2019) 34:995–1003. 10.1111/jdv.16021 31630443

[6] Semenov YR Rosenberg AR Herbosa C Mehta-Shah N Musiek AC. Health-related quality of life and economic implications of cutaneous T-cell lymphoma. Br J Dermatol. (2019) 182:190–6. 10.1111/bjd.17941 30920642 PMC7024588

[7] Jonak C Porkert S Oerlemans S Papadavid E Molloy K Lehner-Baumgartner E. Health-related quality of life in cutaneous lymphomas: past, present and future. Acta Derm Venereol. (2019) 99:640–6. 10.2340/00015555-3171 30868169

[8] Mourad A Gniadecki R. Overall survival in mycosis fungoides: a systematic review and meta-analysis. J Invest Dermatol. (2020) 140:495–7.e5. 10.1016/j.jid.2019.07.712 31465745

[9] Jawed SI Myskowski PL Horwitz S Moskowitz A Querfeld C. Primary cutaneous T-cell lymphoma (mycosis fungoides and Sézary syndrome): part II prognosis, management, and future directions. J Am Acad Dermatol. (2014) 70:223.e1–17. 10.1016/j.jaad.2013.08.033 24438970

[10] Trautinger F Eder J Assaf C Bagot M Cozzio A Dummer R. European organisation for research and treatment of cancer consensus recommendations for the treatment of mycosis fungoides/Sezary syndrome - update 2017. Eur J Cancer. (2017) 77:57–74. 10.1016/j.ejca.2017.02.027 28365528

[11] Prince HM Kim YH Horwitz S Dummer R Scarisbrick J Quaglino P. Brentuximab vedotin or physician's choice in CD30-positive cutaneous T-cell lymphoma (ALCANZA): an international, open-label, randomised, phase 3, multicentre trial. Lancet. (2017) 390:555–66. 10.1016/S0140-6736(17)31266-7 28600132

[12] Duvic M Kuzel TM Olsen EA Martin AG Foss FM Kim YH. Quality-of-life improvements in cutaneous T-cell lymphoma patients treated with denileukin diftitox (ONTAK). Clin Lymphoma. (2002) 2:222–8. 10.3816/CLM.2002.n.003 11970761

[13] Illidge T Chan C Counsell N Morris S Scarisbrick J Gilson D. Phase II study of gemcitabine and bexarotene (GEMBEX) in the treatment of cutaneous T-cell lymphoma. Br J Cancer. (2013) 109:2566–73. 10.1038/bjc.2013.616 24136145 PMC3833210

[14] Talpur R Demierre MF Geskin L Baron E Pugliese S Eubank K. Multicenter photopheresis intervention trial in early-stage mycosis fungoides. Clin Lymphoma Myeloma Leuk. (2011) 11:219–27. 10.1016/j.clml.2011.03.003 21575927

[15] Quaglino P Pimpinelli N Berti E Calzavara-Pinton P Alfonso Lombardo G Rupoli S. Time course, clinical pathways, and long-term hazards risk trends of disease progression in patients with classic mycosis fungoides: a multicenter, retrospective follow-up study from the Italian group of cutaneous lymphomas. Cancer. (2012) 118:5830–9. 10.1002/cncr.27627 22674564

[16] Ling TC Clayton TH Crawley J Exton LS Goulden V Ibbotson S. British association of Dermatologists and British photodermatology group guidelines for the safe and effective use of psoralen-ultraviolet A therapy 2015. Br J Dermatol. (2016) 174:24–55. 10.1111/bjd.14317 26790656

[17] Gallini A Misery L Paul C Le Maître M Aractingi S Joly P Carcinogenic risks of Psoralen UV-A therapy and Narrowband UV-B therapy in chronic plaque psoriasis: a systematic literature review. J Eur Acad Dermatology Venereol. (2012) 26:22–31. 10.1111/j.1468-3083.2012.04520.x 22512677

[18] Stern RS. The risk of melanoma in association with long-term exposure to PUVA. J Am Acad Dermatol. (2001) 44:755–61. 10.1067/mjd.2001.114576 11312420

[19] Stern RS Nichols KT Väkevä LH. Malignant melanoma in patients treated for psoriasis with methoxsalen (psoralen) and ultraviolet a radiation (PUVA). N Engl J Med. (1997) 336:1041–5. 10.1056/NEJM199704103361501 9091799

[20] Vieyra-Garcia P Fink-Puches R Porkert S Lang R Pöchlauer S Ratzinger G Evaluation of low-dose, low-frequency oral Psoralen-UV-A treatment with or without maintenance on early-stage mycosis fungoides: a randomized clinical trial. JAMA Dermatol. (2019) 155:538–47. 10.1001/jamadermatol.2018.5905 30892603 PMC6506892

[21] Duvic M Hymes K Heald P Breneman D Martin AG Myskowski P. Bexarotene is effective and safe for treatment of refractory advanced-stage cutaneous T-cell lymphoma: multinational phase II-III trial results. J Clin Oncol. (2001) 19:2456–71. 10.1200/JCO.2001.19.9.2456 11331325

[22] Duvic M Martin AG Kim Y Olsen E Wood GS Crowley CA Phase 2 and 3 clinical trial of oral bexarotene (Targretin capsules) for the treatment of refractory or persistent early-stage cutaneous T-cell lymphoma. Arch Dermatol. (2001) 137:581–93. 11346336

[23] Stadler R Otte HG Luger T Henz BM Kühl P Zwingers T. Prospective randomized multicenter clinical trial on the use of interferon α-2a plus acitretin versus interferon α-2a plus PUVA in patients with cutaneous T-cell lymphoma stages I and II. Blood. (1998) 92:3578–81. 10.1182/blood.V92.10.3578 9808550

[24] Phan K Ramachandran V Fassihi H Sebaratnam DF. Comparison of narrowband UV-B with Psoralen-UV-A phototherapy for patients with early-stage mycosis fungoides: a systematic review and meta-analysis. JAMA Dermatol. (2019) 155:335–41. 10.1001/jamadermatol.2018.5204 30698622 PMC6439931

[25] Holahan HM Farah RS Fitz S Mott SL Ferguson NN Mckillip J. Health-related quality of life in patients with cutaneous T-cell lymphoma? Int J Dermatol. (2018) 57:1314–9. 10.1111/ijd.14132 30074622

[26] Guitart J. Psoralen plus UV-A therapy in the 21st century. JAMA Dermatol. (2019) 155:529–31. 10.1001/jamadermatol.2018.5844 30892578

[27] Dixon JR. The International conference on harmonization good clinical practice guideline. Qual Assur. (1999) 6:65–74. 10.1080/105294199277860 10386329

[28] Williams JR Public health classics. Bulletin of the World Health Oranization. (2008) 86:650–2. 10.2471/BLT.08.050955 PMC2649471 18797627

[29] Mart E Arias-santiago S Valenzuela-salas I Garrido-colmenero C Dermatology F Quality L Quality of life in persons living with psoriasis patients. J Am Acad Dermatol. (2014) 302–7. 10.1016/j.jaad.2014.03.039 24836080

[30] Patel KR Singam V Vakharia PP Chopra R Sacotte R Patel N. Measurement properties of three assessments of burden used in atopic dermatitis in adults. Br J Dermatol. (2019) 180:1083–9. 10.1111/bjd.17243 30246360 PMC6431274

[31] Heald P Mehlmauer M Martin AG Crowley CA Yocum RC Reich SD Topical bexarotene therapy for patients with refractory or persistent early-stage cutaneous T-cell lymphoma: results of the phase III clinical trial. J Am Acad Dermatol. (2003) 49:801–15. 10.1016/S0190-9622(03)01475-0 14576658

[32] Rencz F Gulácsi L Péntek M Szegedi A Remenyik É Bata-Csörgo Z. DLQI-R scoring improves the discriminatory power of Dermatology life quality index in psoriasis, pemphigus and morphea patients. Br J Dermatol. (2019) 182:1167–75. 10.1111/bjd.18435 31419310

[33] Gahalaut P Mishra N Soodan PS Rastogi MK. Effect of oral puvasol on the quality of life in indian patients having chronic plaque psoriasis. Dermatol Res Pract. (2014) 2014:291586. 10.1155/2014/291586 25276121 PMC4167947

[34] Strober B Papp KA Lebwohl M Reich K Paul C Blauvelt A. Clinical meaningfulness of complete skin clearance in psoriasis. J Am Acad Dermatol. (2016) 75:77–82.e7. 10.1016/j.jaad.2016.03.026 27206759

[35] Norlin JM Nilsson K Persson U Schmitt-Egenolf M. Complete skin clearance and Psoriasis area and severity index response rates in clinical practice: predictors, health-related quality of life improvements and implications for treatment goals. Br J Dermatol. (2020) 182:965–73. 10.1111/bjd.18361 31325318

[36] Väkevä L Niemelä S Lauha M Pasternack R Hannuksela-Svahn A Hjerppe A. Narrowband ultraviolet B phototherapy improves quality of life of psoriasis and atopic dermatitis patients up to 3 months: results from an observational multicenter study. Photodermatol Photoimmunol Photomed. (2019) 35:332–8. 10.1111/phpp.12479 31063610

[37] Simpson EL Gadkari A Worm M Soong W Blauvelt A Eckert L. Dupilumab therapy provides clinically meaningful improvement in patient-reported outcomes (PROs): a phase IIb, randomized, placebo-controlled, clinical trial in adult patients with moderate to severe atopic dermatitis (AD). J Am Acad Dermatol. (2016) 75:506–15. 10.1016/j.jaad.2016.04.054 27268421

[38] Simpson EL Bieber T Eckert L Wu R Ardeleanu M Graham NMH. Patient burden of moderate to severe atopic dermatitis (AD): insights from a phase 2b clinical trial of dupilumab in adults. J Am Acad Dermatol. (2016) 74:491–8. 10.1016/j.jaad.2015.10.043 26777100

[39] Zigmond AS Snaith RP. The hospital anxiety and depression scale. Acta Psychiatr Scand. (1983) 67:361–70. 10.1111/j.1600-0447.1983.tb09716.x 6880820

[40] Marron SE Tomas-aragones L Boira S. Anxiety, depression, quality of life and patient satisfaction in Acne patients treated with oral Isotretinoin. Acta Derm Venereol. (2013) 93:701–6. 10.2340/00015555-1638 23727704

[41] Shyu Y Firth J Koyanagi A Solmi M Alavi A Piguet V. Depression and anxiety in adults with Hidradenitis suppurativa a systematic review and meta-analysis. JAMA Dermatol. (2019) 155:939–45. 10.1001/jamadermatol.2019.0759 31166590 PMC6551580

[42] Ring J Zink A Arents BWM Seitz IA Mensing U Schielein MC. Atopic eczema: burden of disease and individual suffering – results from a large EU study in adults. J Eur Acad Dermatol Venereol. (2019) 33:1331–40. 10.1111/jdv.15634 31002197

[43] Silverberg JID Gelfand JM Margolis DJ Boguniewicz M Fonacier L Grayson MH. Symptoms and diagnosis of anxiety and depression in atopic dermatitis in U. S. adults. Br J Dermatol. (2019) 181:554-6. 10.1111/bjd.17683 30838645 PMC6850653

[44] Modalsli EH Åsvold BO Snekvik I Romundstad PR Naldi L Saunes M The association between the clinical diversity of psoriasis and depressive symptoms: the HUNT study, Norway. J Eur Acad Dermatology Venereol. (2017) 31:2062–8. 10.1111/jdv.14449 28662282

[45] Yuan J Ding R Wang L Sheng L Li J Hu D. Screening for depression in acute coronary syndrome patients: a comparison of patient health questionnaire-9 versus hospital anxiety and depression scale-depression. J Psychosom Res. (2019) 121:24–8. 10.1016/j.jpsychores.2019.03.018 30928210

[46] Łabuz-Roszak B Niewiadomska E Kubicka-Baczyk K Skrzypek M Dobrakowski P Tyrpien-Golder K. Prevalence of pain in patients with multiple sclerosis and its association with anxiety, depressive symptoms and quality of life. Psychiatr Pol. (2019) 53:475–86. 10.12740/PP/94469 31317971

[47] Ivziku D Clari M Piredda M De Marinis MG Matarese M. Anxiety, depression and quality of life in chronic obstructive pulmonary disease patients and caregivers: an actor–partner interdependence model analysis. Qual Life Res. (2019) 28:461–72. 10.1007/s11136-018-2024-z 30341578

[48] Almeida E De Simone M Assessment of the Hospital Anxiety and Depression Scale (HADS) performance for the diagnosis of anxiety in patients with systemic lupus erythematosus. Rheumatol Int. (2017) 37:1999–2004. 10.1007/s00296-017-3819-x 28940018

[49] Chan CYY Tsang HHL Lau CS Chung HY. Prevalence of depressive and anxiety disorders and validation of the Hospital anxiety and depression scale as a screening tool in axial spondyloarthritis patients. Int J Rheum Dis. (2017) 20:317–25. 10.1111/1756-185X.12456 25293872

[50] Wagner T Augustin M Blome C Forschner A Garbe C Gutzmer R. Fear of cancer progression in patients with stage IA malignant melanoma. Eur J Cancer Care. (2018) 27:e12901. 10.1111/ecc.12901 30126009

[51] Licari A Ciprandi R Marseglia G Ciprandi G Behavioral sciences Anxiety and depression in Adolescents with severe asthma and in their parents : preliminary results after 1 year of treatment. Behav Sci. (2019) 9:78 10.3390/bs9070078 PMC6680478 31337076

[52] Tesio V Marra S Molinaro S Torta R Gaita F Castelli L. Screening of depression in cardiology: a study on 617 cardiovascular patients. Int J Cardiol. (2017) 245:49–51. 10.1016/j.ijcard.2017.07.065 28747268

[53] Preljevic VT Østhus TBH Sandvik L Opjordsmoen S Nordhus IH Os I. Screening for anxiety and depression in dialysis patients: comparison of the hospital anxiety and depression scale and the beck depression inventory. J Psychosom Res. (2012) 73:139–44. 10.1016/j.jpsychores.2012.04.015 22789418

[54] Jozic I Stojadinovic O Kirsner RSF Tomic-Canic M. Skin under the (Spot)-light: Cross-talk with the central hypothalamic-pituitary-adrenal (HPA) axis. J Invest Dermatol. (2015) 135:1469–71. 10.1038/jid.2015.56 25964265

[55] Fell GL Robinson KC Mao J Woolf CJ Fisher DE. Skin β-endorphin mediates addiction to UV light. Cell. (2014) 157:1527–34. 10.1016/j.cell.2014.04.032 24949966 PMC4117380

[56] Tejeda HA Bonci A. Shedding “uV” light on endogenous opioid dependence. Cell. (2014) 157:1500–1. 10.1016/j.cell.2014.06.009 24949960 PMC9840560

[57] Bigliardi PL Dancik Y Neumann C Bigliardi-Qi M. Opioids and skin homeostasis, regeneration and ageing – what's the evidence? Exp Dermatol. (2016) 25:586–91. 10.1111/exd.13021 27060353

[58] Felton SJ Kendall AC Almaedani AFM Urquhart P Webb AR Kift R. Serum endocannabinoids and N-acyl ethanolamines and the influence of simulated solar UVR exposure in humans *in vivo*. Photochem Photobiol Sci. (2017) 16:564–74. 10.1039/C6PP00337K 28138687

