# Supplementary material for: Quality of Life, Anxiety, and Depression in Patients With Early-Stage Mycosis Fungoides and the Effect of Oral Psoralen Plus UV-A (PUVA) Photochemotherapy on it
Source: Front Med (Lausanne). 2020 Aug 5;7:330. doi: 10.3389/fmed.2020.00330 (PMC7419471; doi:10.3389/fmed.2020.00330)
Supplement: Supplementary file 1 [file Data_Sheet_1.pdf]

Supplementary documents

Supplemental Methods: Items of the study questionnaires (DLQI and HADS)

Supplemental figure 1: Study chart and availability of questionnaires

Supplemental figure 2: Development of DLQI impairment and HADS-D and HADS-A severity categories

Supplemental figure 3: Correlation and regression of QoL impairment, psychological discomfort and mSWAT

Supplemental figure 4: Evolution of HADS items

Supplemental table 1: Subgroup analysis of DLQI, HADS-A and HADS-D for complete and partial responders

Supplemental table 2: Robustness of quality of life improvement

Supplemental table 3: Subgroup analysis of DLQI, HADS-A and HADS-D for patients in maintenance or observance arm

Supplemental table 4: Correlation of QoL impairment, psychological discomfort and mSWAT

Dermatology Life Quality Index (DLQI)

1. Over the last week, how itchy, sore, painful or stinging has your skin been?
2. Over the last week, how embarrassed or self-conscious have you been because of your skin?
3. Over the last week, how much has your skin interfered with you going shopping or looking after your home or garden?
4. Over the last week, how much has your skin influenced the clothes you wear?
5. Over the last week, how much has your skin affected any social or leisure activities?
6. Over the last week, how much has your skin made it difficult for you to do any sport?
7. Over the last week, has your skin prevented you from working or studying? If "No", over the last week how much has your skin been a problem at work or studying?
8. Over the last week, how much has your skin created problems with your partner or any of your close friends or relatives?
9. Over the last week, how much has your skin caused any sexual difficulties?
10. Over the last week, how much of a problem has the treatment for your skin been, for example by making your home messy, or by taking up time?

Patients can award each item with 0 points (no impairment); 1 (slight), 2 (moderate) and 3 points (strong impairment) or mark items as not relevant.

Hospital Anxiety and Depression Scale (HADS-A and HADS-D)

Anxiety (HADS-A)

1. I feel tense or 'wound up'
2. I get a sort of frightened feeling as if something awful is about to happen
3. Worrying thoughts go through my mind
4. I can sit at ease and feel relaxed
5. I get a sort of frightened feeling like 'butterflies' in the stomach
6. I feel restless as I have to be on the move
7. I get sudden feelings of panic

Depression (HADS-D)

1. I still enjoy the things I used to enjoy
2. I can laugh and see the funny side of things
3. I feel cheerful
4. I feel as if I am slowed down
5. I have lost interest in my appearance
6. I look forward with enjoyment to things
7. I can enjoy a good book or radio or TV program

Patients can award each item with 0 points (no impairment); 1 (slight), 2 (moderate) and 3 points (strong impairment).

Methods: Items of the study questionnaires (DLQI and HADS)

Items questioned in the Dermatology Life Quality Index (DLQI) and Hospital Anxiey and Depression Scale (HADS-A & HADS-D).

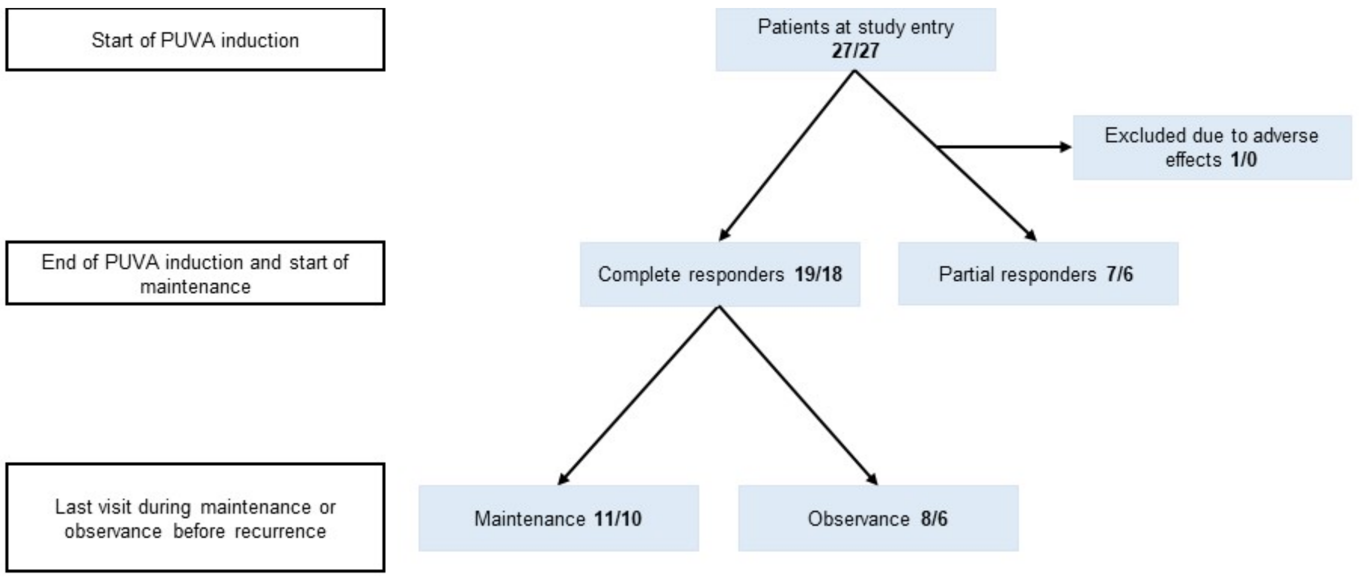

Supplemental figure 1: Study chart and availability of questionnaires
Numbers in boxes indicate patients in the study/number of patients with completed questionnaires (Dermatology Life Quality Index, Hospital Anxiety and Depression Scale) for the difference phases of the study.

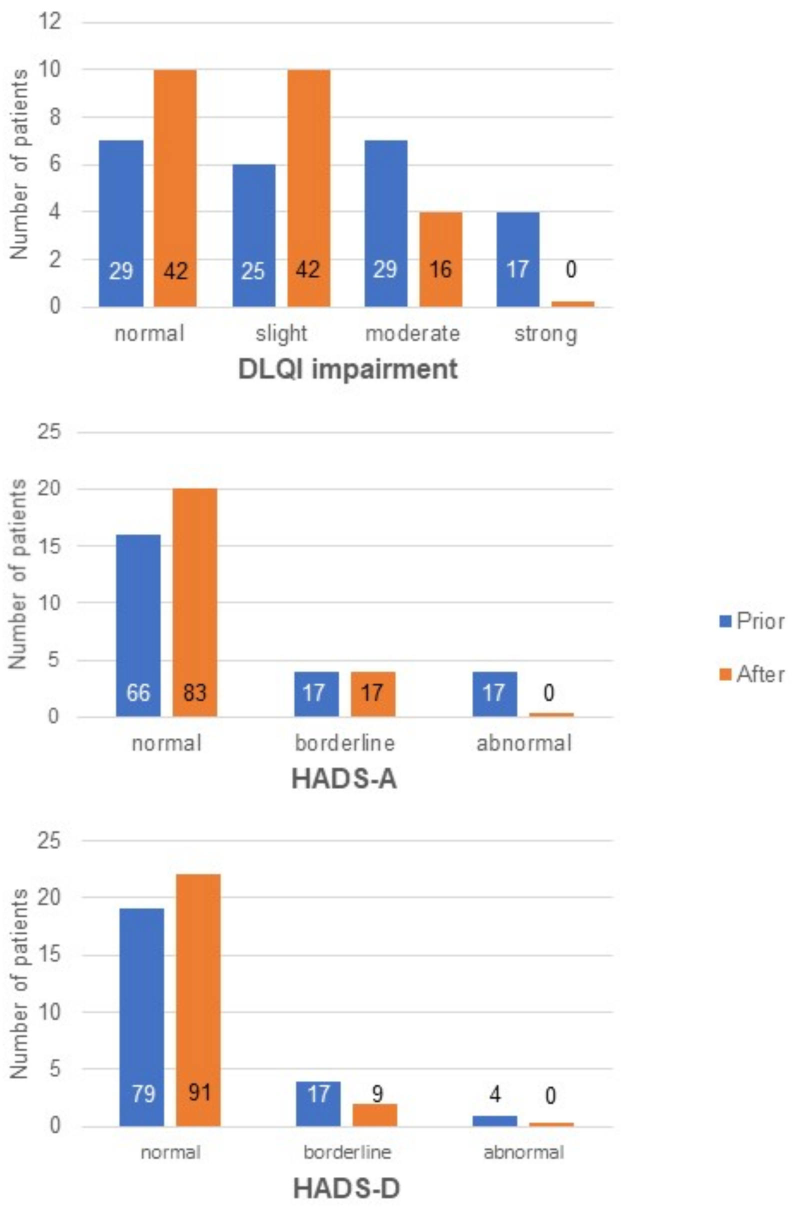

Supplemental figure 2: Development of DLQI impairment and HADS-D and HADS-A severity categories

Number of patients and their percentages (plotted inside or near the bars) in a certain category of impairment/affection in Dermatology Life Quality Index (DLQI) (normal, 0-1 points; slight, 2-5; moderate, 6-10 and strong, >10 points) and Hospital Anxiety and Depression Scale (HADS-A & HADS-D) (normal, 0-7 points; borderline abnormal, 8-10 and abnormal, >10 points). Severity categories are depicted for the timepoints prior and after PUVA induction treatment (orange/blue).

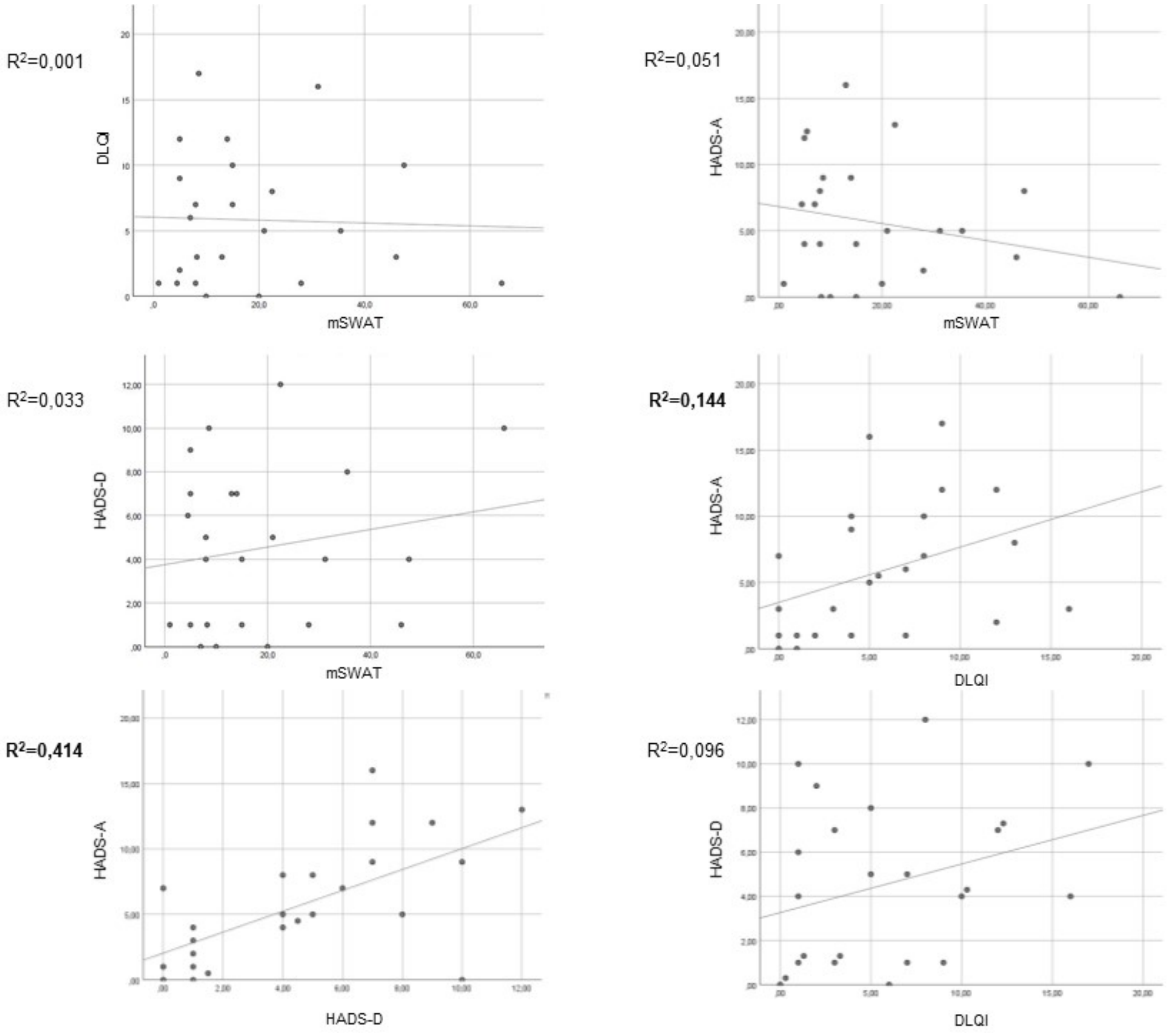

Supplemental figure 3: Correlation and regression of QoL impairment, psychological discomfort and mSWAT:

Scatterplots for Spearman analysis of correlation and regression of Dermatology Life Quality Index (DLQI), Hospital Anxiety and Depression Scale (HADS-A & HADS-D) and modified Severity-Weighted Assessment Tool (mSWAT) prior to psoralen-UV-A (PUVA) induction. Regression lines are shown and values for goodness of fit (R^2) are plotted in the top left corner. Bold numbers represent significant correlation.

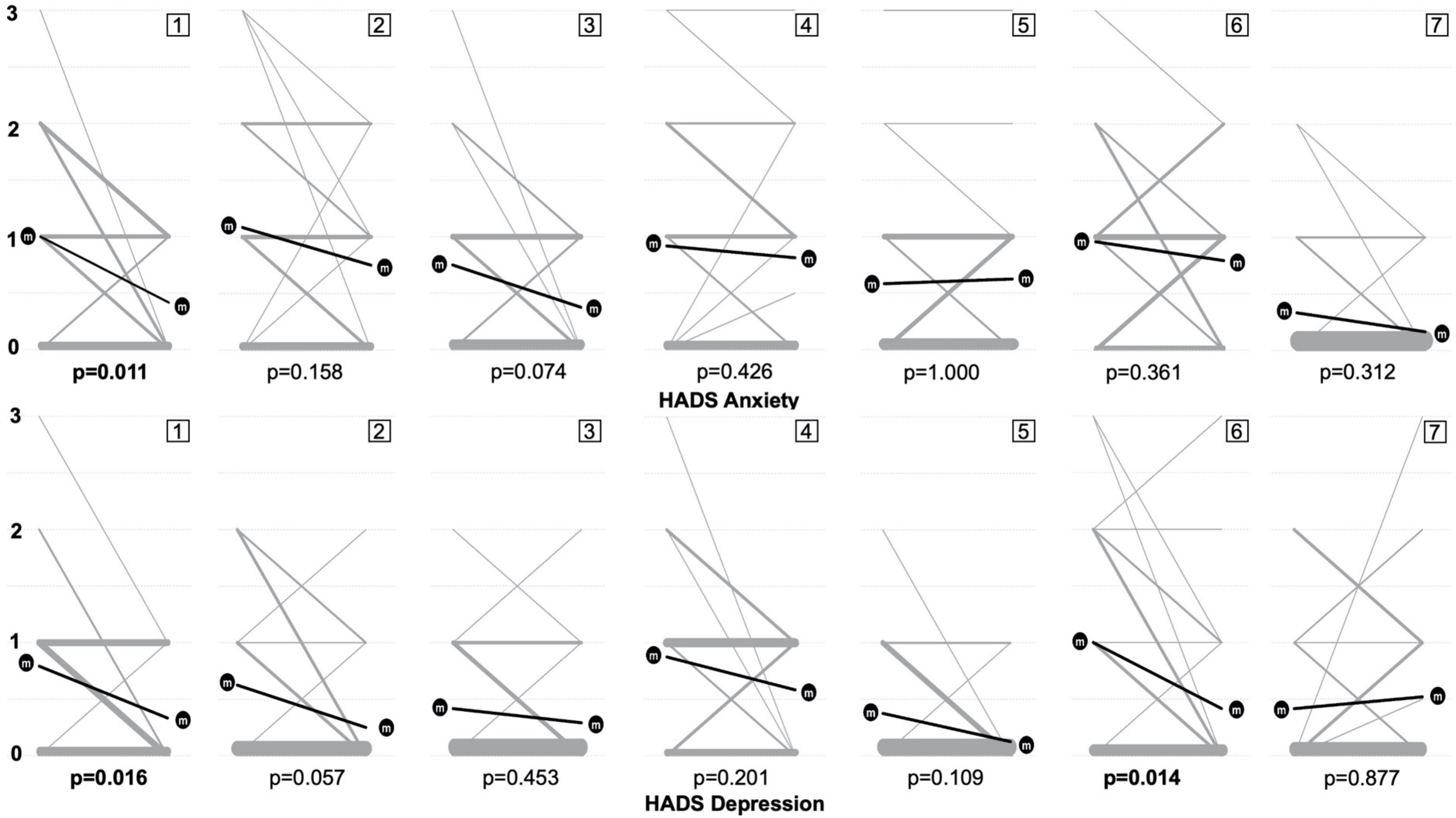

Supplemental figure 4: Evolution of HADS items

Individual values of the 14 Hospital Anxiety and Depression (HADS-A & HADS-D) scale for the timepoints prior to (left side) and after (right side) Psoralen-UV-A (PUVA) treatment. Upper panel shows items of HADS-A lower panels HADS-D. Numbers in square boxes on top of the panels represent item number of HADS-A and HADS-D: HADS-A item 1 (inner tension), item 2 (frightened feelings), item 3 (worrying thoughts), item 4 (feeling relaxed), item 5 (frightened feeling), item 6 (restlessness) and item 7 (feeling of panic); HADS-D item 1 (enjoyment of things); item 2 (seeing the funny side of things); item 3 (feeling cheerful), item 4 (feeling slowed down); item 5 (interest in appearance), item 6 (looking forward with enjoyment), item 7 (enjoying things). Each item can be awarded with 0 to 3 points. Thickness of lines is proportional to the number of patients and their HADS evoluting in a certain way. Statistical comparison was performed by Wilcoxon-signed-rank-test and respective p-values are shown underneath plots. Evolution of mean value (m) is shown by the black line and black circles.

	Complete Responders		Partial Responders		Difference		
	Prior to therapy	End of induction	Prior to therapy	End of induction	Complete responders	Partial responders	p-value
**DLQI**	5.33±4.91	2.00±1.94	7.33±5.53	3.67±4.03	-3.33 (62.5%)	-3.66 (49.9%)	0.708
**HADS A**	5.67±4.76	3.86±2.65	5.50±4.32	4.16±3.60	-1.81 (31.9%)	-1.34 (24.4%)	0.933
**HADS D**	4.16±3.57	1.97±1.87	5.50±4.04	4.16±4.02	-2.19 (52.6%)	-1.34 (24.4%)	0.634

Supplemental table I: Subgroup analysis of DLQI, HADS-A and HADS-D for complete and partial responders

Subgroup analysis of Dermatology Life Quality Index (DLQI) and Hospital Anxiety and Depression Scale (HADS-A and HADS-D) in complete (n=18) and partial responders (n=6) comparing values prior and after psoralen-UV-A (PUVA) induction. Difference was calculated by subtracting individual score values at the end of induction treatment from those prior to therapy. Data shown are means and standard deviations. Numbers in parentheses represent percentage of reduction. Statistical significance was tested using Mann-Whitney-U test.

	DLQI all items	DLQI - item 1	DLQI - item 1+2	DLQI - item 1+2+3
**p-value**	0.003	0.012	0.012	0.039

Supplemental table II: Robustness of quality of life improvement:

Table contains p-values comparing overall scores prior to and after psoralen-UV-A (PUVA) induction with all Dermatology Life Quality Index (DLQI) items (1-10) and without significantly improved items (1-3) in the single item analysis (Figure 1) using Wilcoxon-signed-rank-test. Item 1: itching, stinging, aching; item 2: embarrassment/self-conscious; item 3: skin interfering with shopping, gardening, looking after home.

	Maintenance therapy			Observance			Difference (induction end – last observation)		
	Start of induction	End of induction	End of maintenance	Start of induction	End of induction	End of observance	Maintenance	Observance	p-value
**DLQI**	5.22±4.17	1.56±1.24	1.78±2.44	6.86±5.87	3.00±2.51	3.57±2.89	0.22 (8.5%)	0.57 (8.3%)	0.351
**HADS A**	4.78±4.68	3.67±2.91	3.44±3.43	7.43±4.96	5.00±1.91	5.43±4.76	-0.23 (-4.8%)	0.43 (5.7%)	0.895
**HADS D**	4.11±4.60	2.22±2.33	1.44±2.00	4.57±2.07	1.86±1.46	3.71±5.94	-0.78 (-19%)	1.85 (40.5%)	0.644

Supplemental table III: Subgroup analysis of DLQI, HADS-A and HADS-D for patients in maintenance or observance arm

Subgroup analysis of Dermatology Life Quality Index (DLQI) and Hospital Anxiety and Depression Scale (HADS-A and HADS-D) in patients receiving Psoralen-UV-A (PUVA) maintenance therapy or not (observance). Values obtained at the end of PUVA induction and at the last visit of maintenance or at the last visit before recurrence within 9 months were compared for the patients allocated to the maintenance (n=10) or observance arm (n=6). Difference was calculated by subtracting individual score values at the end of maintenance or observance phase (within 9 months) from those at the end of PUVA induction. Data shown are means and standard deviations. Numbers in parentheses represent percentage of reduction. Statistical significance was tested using Mann-Whitney-U test.

**Prior to induction**	DLQI	HADS-A	HADS-D	mSWAT
DLQI		0.518 (**0.009**)	0.342 (0.102)	0.083 (0.700)
HADS-A			0.643 (**0.001**)	-0.181 (0.397)
HADS-D				0.111 (0.607)
mSWAT				

Supplemental table IV: Correlation of QoL impairment, psychological discomfort and mSWAT:

Spearman analysis for correlation of Dermatology Life Quality Index (DLQI), Hospital Anxiety and Depression Scale (HADS-A & HADS-D) and modified Severity-Weighted Assessment Tool (mSWAT) with values obtained prior to induction therapy. rho values are plotted; numbers in parentheses are p-values. Significance is set in bold.
